# Correction to ‘Most protein domains exist as variants with distinct functions across cells, tissues and diseases’

**DOI:** 10.1093/nargab/lqad094

**Published:** 2023-10-11

*NAR Genomics and Bioinformatics*, Volume 5, Issue 3, September 2023, lqad084, https://doi.org/10.1093/nargab/lqad084

In the originally published version of this manuscript, the following statement was inadvertently omitted from the Funding section:

‘Funded by a generous grant from the Lundbeck Foundation: R413-2022-878.’

This has been corrected.

