# Supplementary material for: Most protein domains exist as variants with distinct functions across cells, tissues and diseases
Source: NAR Genom Bioinform. 2023 Sep 20;5(3):lqad084. doi: 10.1093/nargab/lqad084 (PMC10516350; doi:10.1093/nargab/lqad084)
Supplement: lqad084_supplemental_files [file lqad084_supplemental_files.zip › Supplementary figures v3.pdf]

Most protein domains exist as variants with distinct functions across cells, tissues, and diseases

Kristoffer Vitting-Seerup

Supplementary Figures

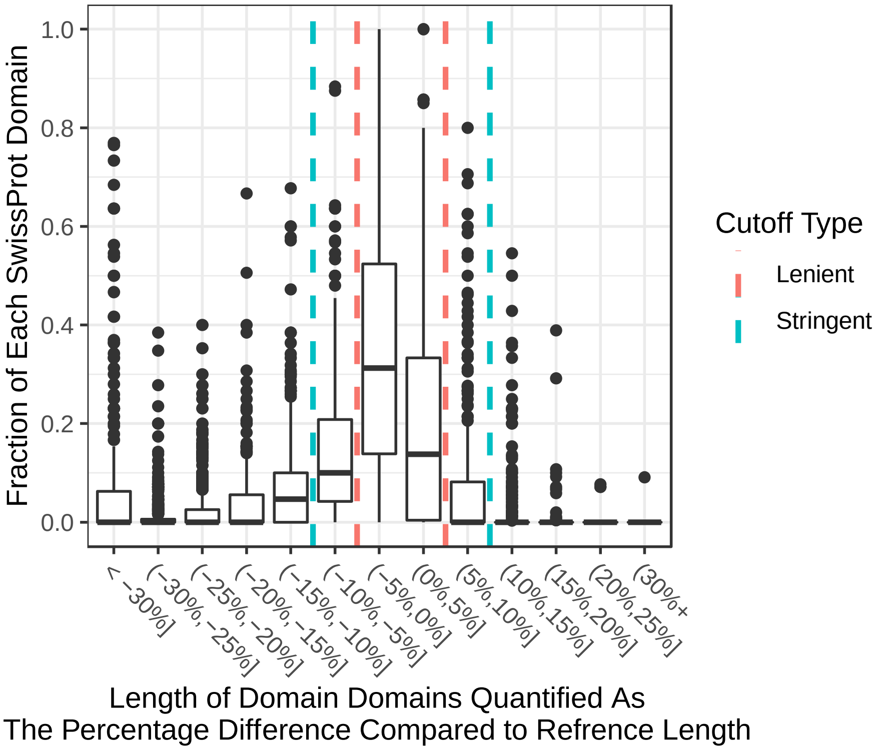

Figure S1. The frequency (y-axis) of the difference between observed protein domain length and the corresponding reference model measured as a fraction of the reference length. The length differences across all domains identified in SwissProt and each bin is shown as a boxplot. Length differences capped at +/- 30% for visual purposes. Stringent and lenient cutoffs for defining isotypes are shown.

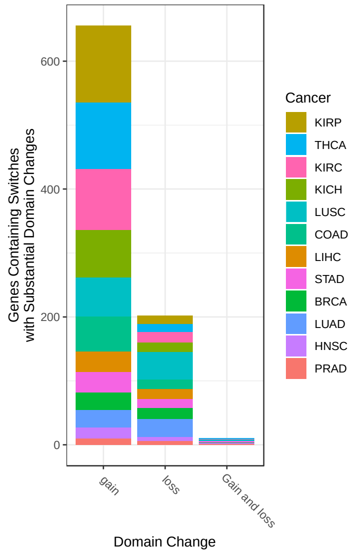

Figure S2. For each cancer type (color), the number of genes with partial gain/loss of protein domains (y-axis). Genes are divided based on whether the isoform switch resulted in a gain, a loss, or both gain/loss of a parts of the protein domain (x-axis). BRCA: Breast invasive carcinoma, COAD: Colon adenocarcinoma, HNSC: Head and Neck squamous cell carcinoma, KICH: Kidney Chromophobe, KIRC: Kidney renal clear cell carcinoma, KIRP: Kidney renal papillary cell carcinoma, LIHC: Liver hepatocellular carcinoma, LUAD: Lung adenocarcinoma, LUSC: Lung squamous cell carcinoma, PRAD: Prostate adenocarcinoma, STAD: Stomach adenocarcinoma, THCA: Thyroid carcinoma.

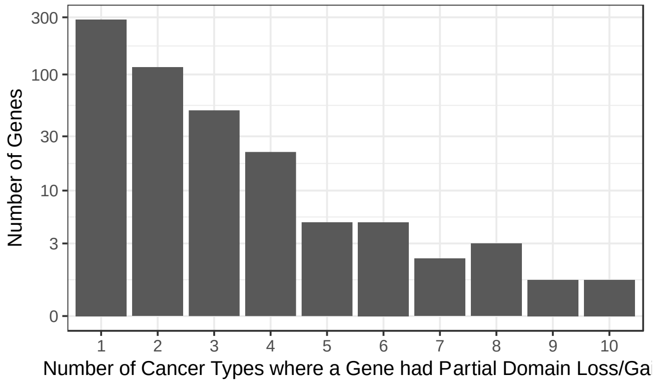

Figure S3. The number of cancers in which each gene from Figure S2 was identified.

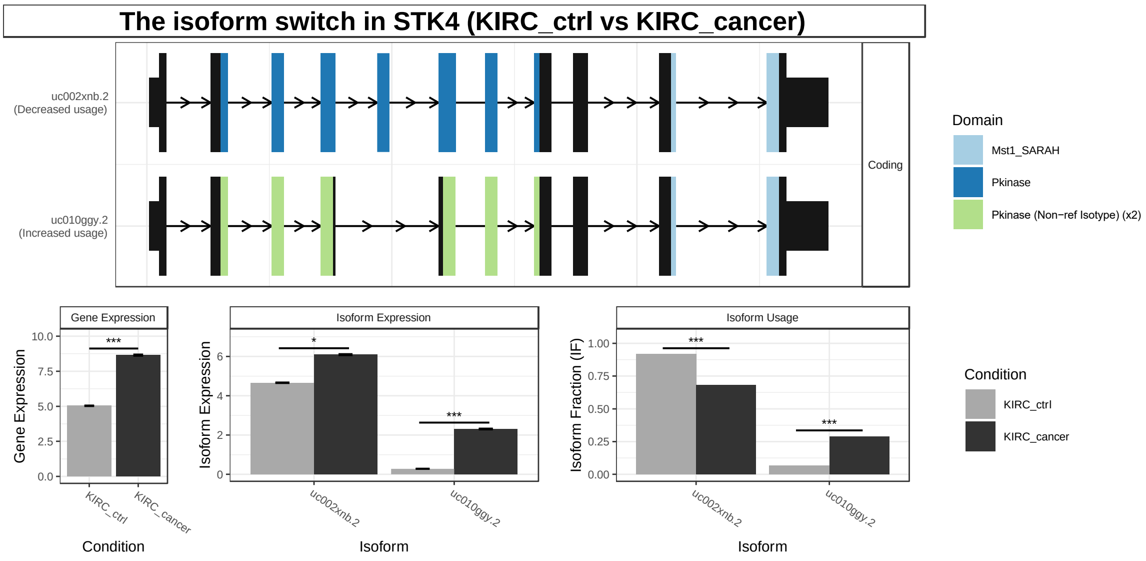

Figure S4. The switchPlot of STK4 in Kidney Renal Clear Cell Carcinoma (KIRC). Top sub-plots as described in Figure 1A of main text. Bottom row of sub-plots shows the conditional gene expression (left), transcript expression (midt) and isoform fraction (right). Isoform fractions indicate how large a fraction each isoform contribute to the overall gene expression. *** denotes FDR corrected p-values < 0.001. * denotes FDR corrected p-values < 0.05

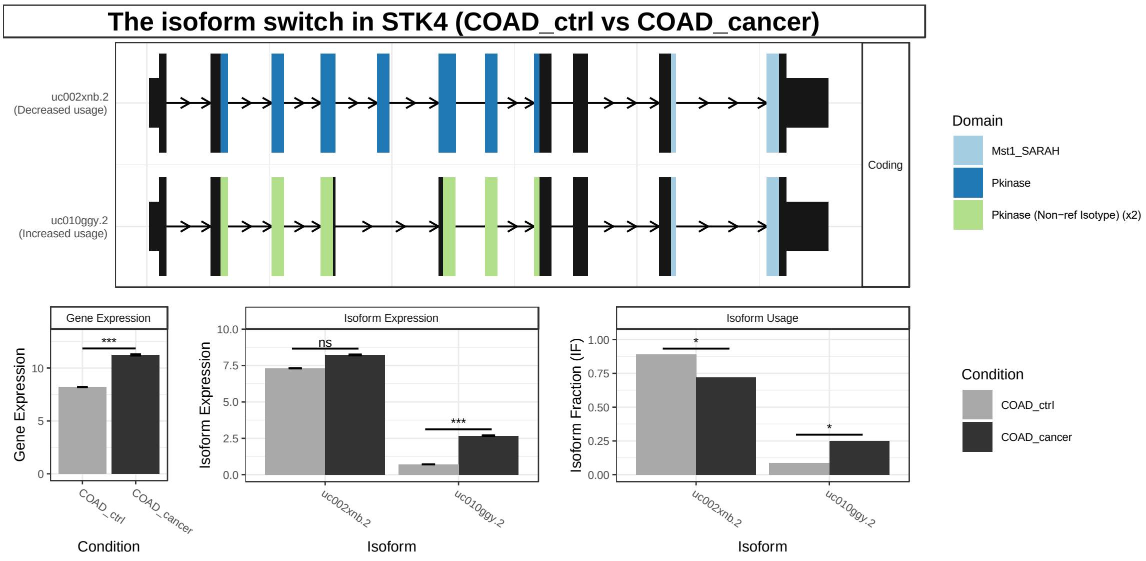

Figure S5. The switchPlot of STK4 in Colon adenocarcinoma (COAD) as described in Figure S4.

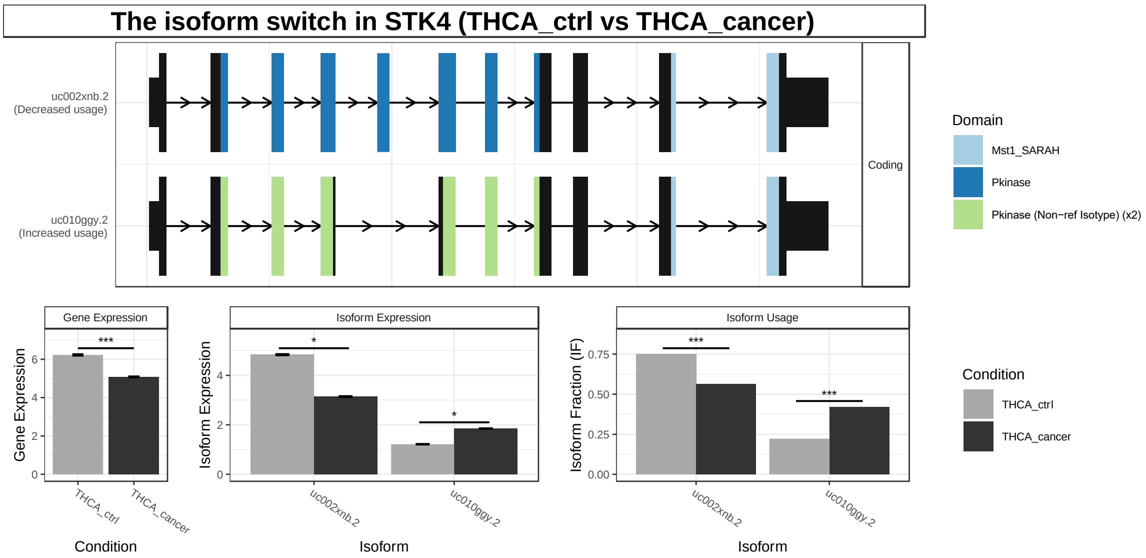

Figure S6. The switchPlot of STK4 in Thyroid carcinoma (THCA) as described in figure S4.

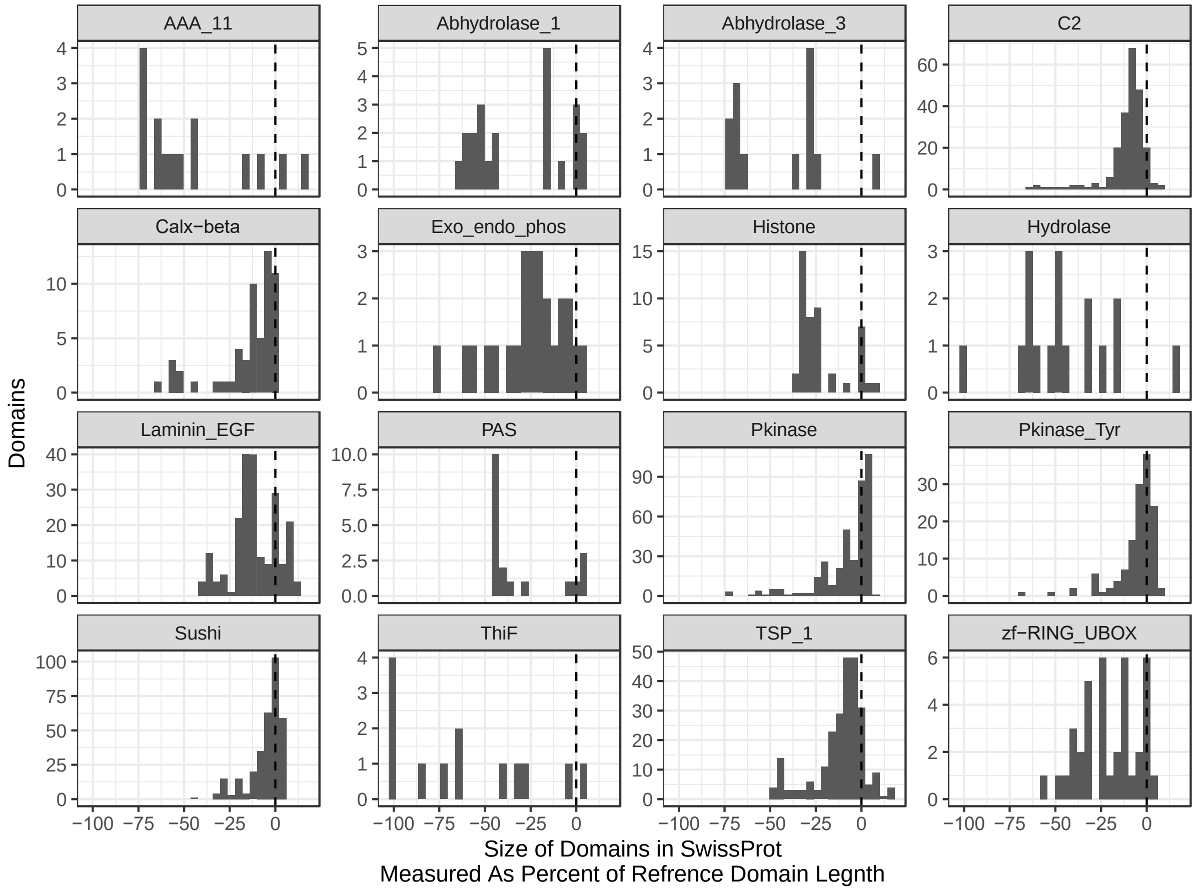

Figure S7. The domain length differences of the 16 protein that most frequently have large domain differences (more extreme than 25% of the reference length). The difference is measured as a fraction of the reference length with the dashed line indicating no difference.

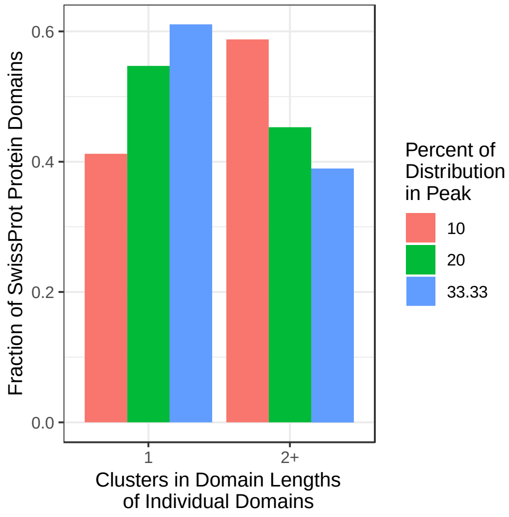

Figure S8. The number of clusters found in the length distribution of each protein domain identified in SwissProt. Color indicate the minimum percent of domain lengths in each cluster.

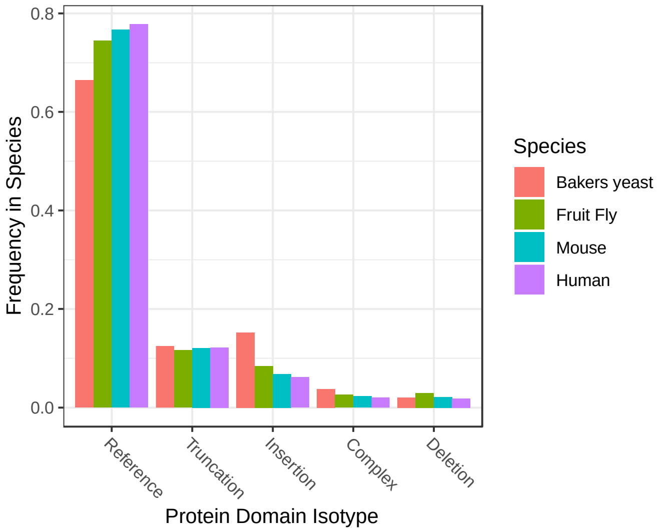

Figure S9. The frequency (y-axis) of each protein domain isotype (x-axis) in SwissProt proteins for different species (color).

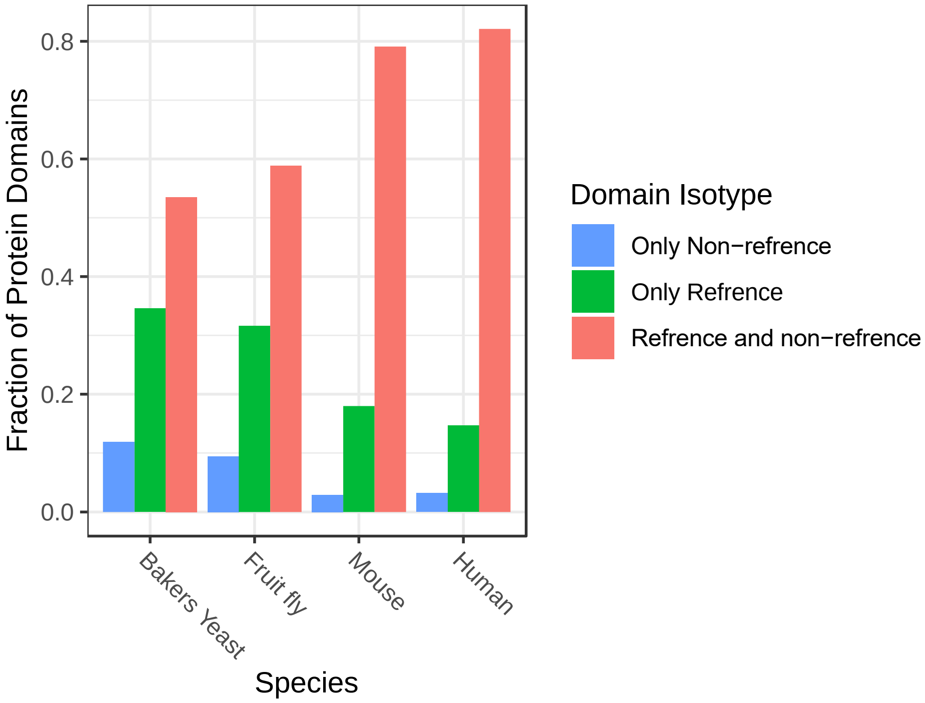

Figure S10. The fraction of protein domains that exist as reference and non-reference isotypes in SwissProt of various species when only considering proteins annotated as "domain containing" in SwissProt.

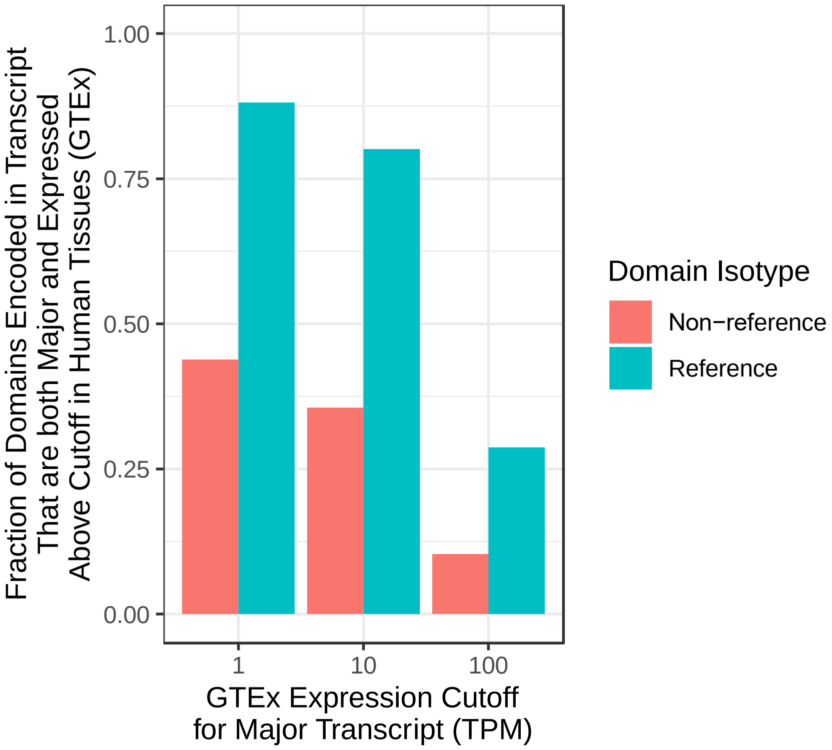

Figure S11. The fraction (Y-axis) of domain isotypes (color) contained in a major transcript also expressed above a Transcript Per Million (TPM) cutoff (x-axis) in at least one human tissue. A major transcript is defined as the transcript that is most expressed from its gene in at least one tissue type.

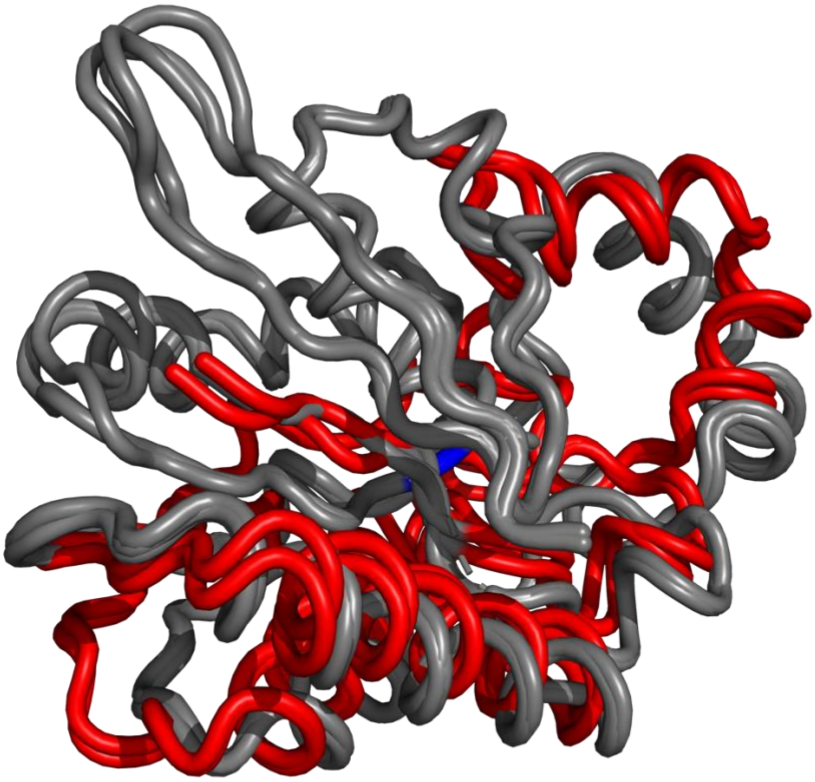

Figure S12. Structural alignment of three references (grey) and two truncated (red) isotypes of the His_Phos_1 protein domain. The catalytic residue His108 is indicated in blue. The same as in the main figure just shown from another angle.

```
                  1        .         .         .         .         .         .        70
1K6M_A (ref)      IYLCRHGESELNIRGRIGG--DSGLSVRGKQYAYALANFIQSQGISSLKVFTSRMKRTIQTAEALGVPYE
5HTK_A (ref)      IYLCR-GESEFNLLGKIGG--DSGLSVRGKQFAQALRKFLEEQEITDLKVWTSQLKRTIQTAESLGVPYE
6HVH_A (ref)      IYLCRHGENEHNLQGRIGG--DSGLSSRGKKFASALSKFVEEQNLKDLRVWTSQLKSTIQTAEALRLPYE
1YFK_A (non-ref)  LVLIRHGESAWNLENRFSGWYDADLSPAGHEEAKRGGQALRDAGYEFDICFTSVQKRAIRTLWTVLDAID
2A9J_A (non-ref)  LIMLRHGEGAWNKENRFCSWVDQKLNSEGMEEARNCGKQLKALNFEFDLVFTSVLNRSIHTAWLILEELG
                  ^ ^ * **   *   ^     *  *   *   *      ^          ^**     * *   ^
                  1        .         .         .         .         .         .        70

                  71        .         .         .         .         .         .        140
1K6M_A (ref)      QFKALNEIDAGVCEEMTYEEIQEHYPEEFALRDQDKYRYRYPKGESYEDLVQRLEPVIMELERQENVLVI
5HTK_A (ref)      QWKILNEIDAGVCEEMTYAEIEKRYPEEFALRDQEKYLYRYPGGESYQDLVQRLEPVIMELERQGNVLVI
6HVH_A (ref)      QWKALNEIDAGVCEELTYEEIRDTYPEEYALREQDKYYYRYPTGESYQDLVQRLEPVIMELERQENVLVI
1YFK_A (non-ref)  QMWLPVVRTWRLNERHYGGLTGLNKAETAAKHGEAQVKIWRRSYDVPPPPMEPDHPFYSNISKDRRYA--
2A9J_A (non-ref)  QEWVPVESSWRLNERHYGALIGLNREQMALNHGEEQVRLWRRSYNVTPPPIEESHPYYQEIYNDRRYKVC
                  *          ^ *                 ^                  ^    *    ^
                  71        .         .         .         .         .         .        140

                  141        .         .         .         .         .         .        210
1K6M_A (ref)      CHQAVMRCLLAYFLDKSSEELPYLKCPLHTVLKLTPVAYGCKVESIYLNVEAVNTHREKPENVDITREPE
5HTK_A (ref)      SHQAVMRCLLAYFLDKGADELPYLRCPLHTIFKLTPVAYGCKVETIKLNVEAVNTHRDKPTNNFPK----
6HVH_A (ref)      CHQAVLRCLLAYFLDKSAEEMPYLKCPLHTVLKLTPVAYGCRVESIYLNVESVCTHRERSE---------
1YFK_A (non-ref)  --------------DLTEDQLPSCESLKDTIARALPFWNEEIVPQIKEGKRVLIAAHGN-----------
2A9J_A (non-ref)  --------------DVPLDQLPRSESLKDVLERLLPYWNERIAPEVLRGKTILISA--------------
                       *   ^ ^*        ^ ^  *         ^      ^
                  141        .         .         .         .         .         .        210

                  211        220
1K6M_A (ref)      EALDTVPAHY
5HTK_A (ref)      ----------
6HVH_A (ref)      ----------
1YFK_A (non-ref)  ----------
2A9J_A (non-ref)  ----------

                  211        220
```

Figure S13. Sequence alignment comparing the structure of 5 His_Phos_1 domains from distinct structures (and genes) shown in figure 2A and S12. The PDB id and chain are given as sequence ids to the left. Domain isotype is given in brackets. The core catalytical residues are in bold, enlarged, and colored red when conserved between isotypes, and blue when missing from the non-reference domain isotype. The grey sequence are the residues aligned when 50 amino acids downstream of the domain boundary are included (note that for the reference isotype this exceeds the end of the protein hence fewer amino acids can be shown in grey).

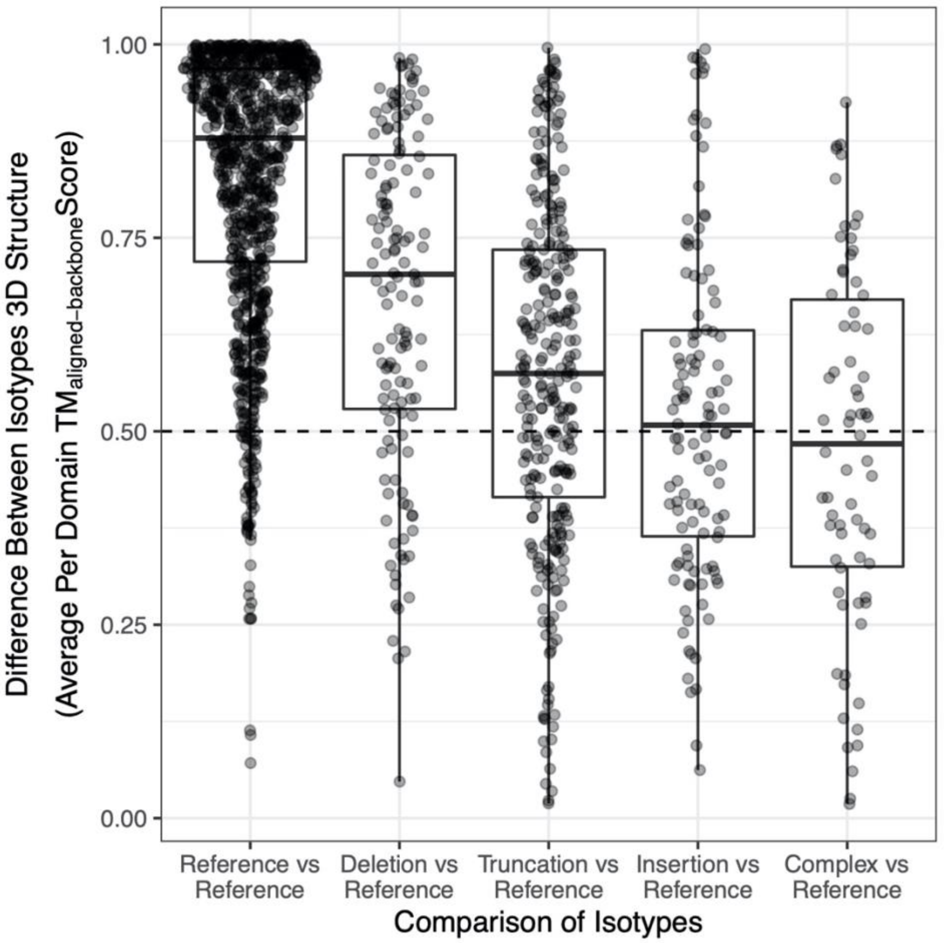

Figure S14. For each protein domain, the 3D structures of reference isotypes were compared to the structure of all analyzed isotypes (x-axis). The structural difference was quantified using TM-scores and averaged per domain (Y-axis) (higher scores mean more similar). The dashed line indicates where higher TM scores is expected to originate form structures in approximately the same fold. Below dashed line suggest different folds.

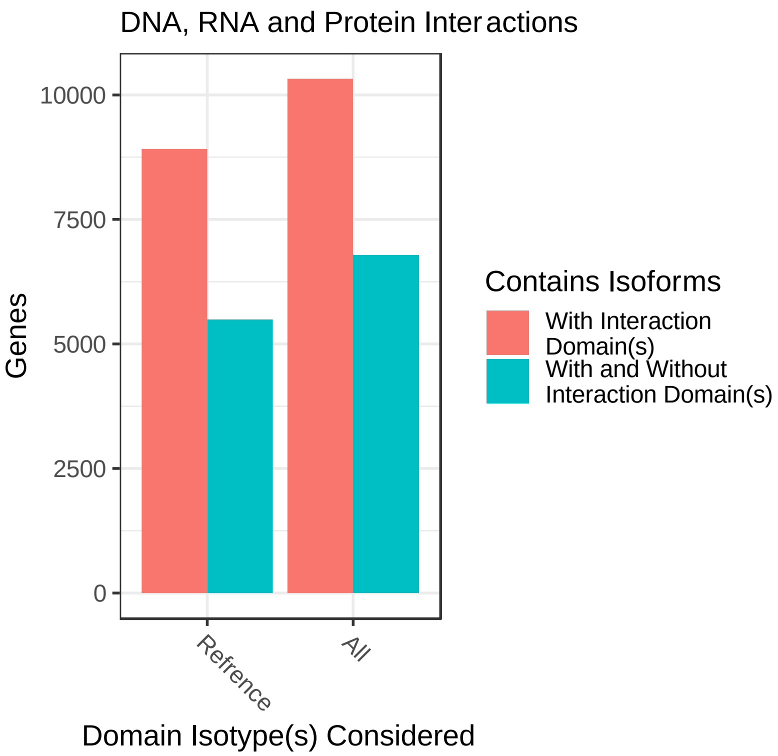

Figure S15. The number of GENCODE genes (y-axis) that has isoforms encoding an interaction domain (red) or isoforms that both encode and does not encode an interaction domain (blue) depending on whether only "reference" isotypes are counted or all isotypes are counted (x-axis).

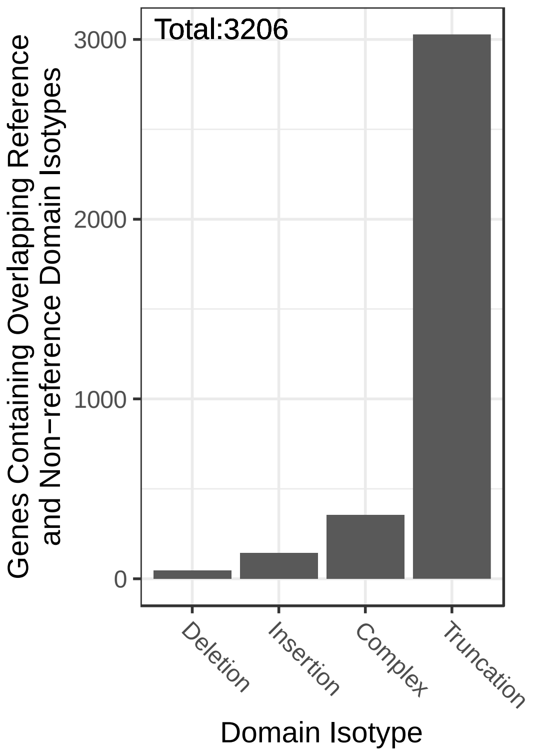

Figure S16. For each isotype (x-axis), the number of GENCODE genes that encodes reference and non-reference domain isotypes from the same genomic region (encoded in different

isoforms)(Y-axis). The total number of genes encoding at least one such pair is indicated in the top left corner.

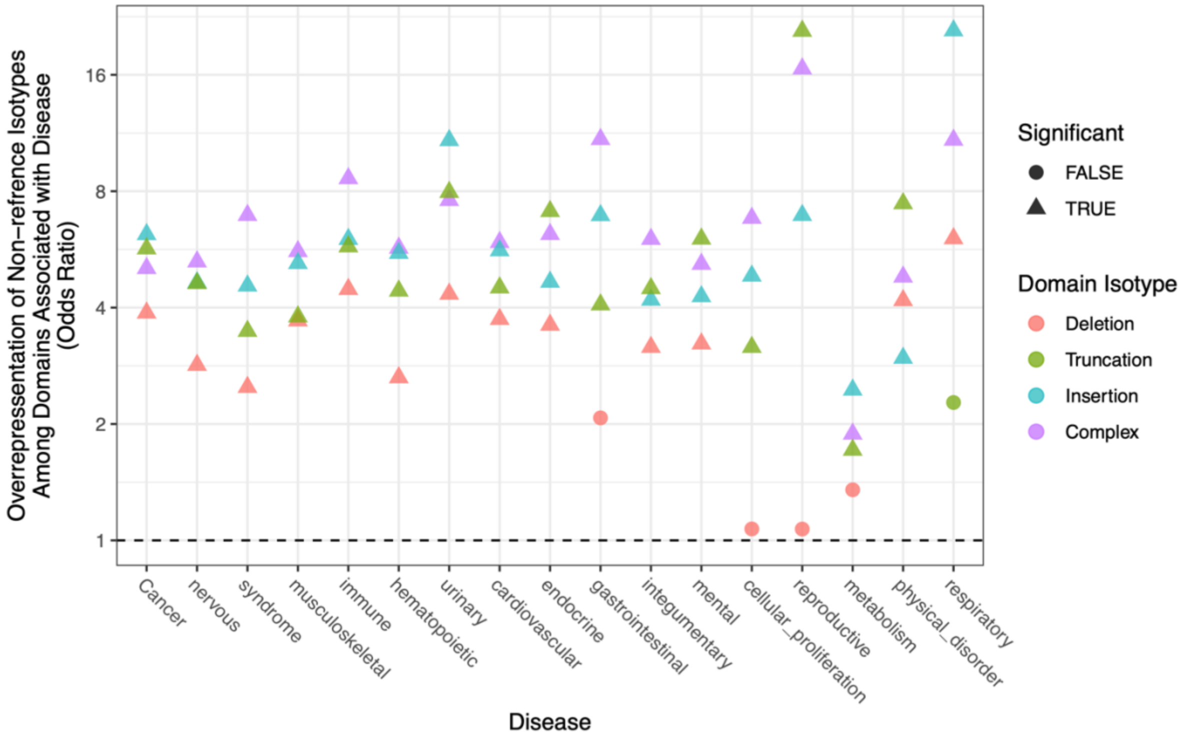

Figure S17. The enrichment (y-axis) of non-reference domain isotypes (color) among domains associated with various disease classes (x-axis). Shape denote FDR < 0.05
